# Genomic Analysis Reveals Human-Mediated Introgression From European Commercial Pigs to Henan Indigenous Pigs

**DOI:** 10.3389/fgene.2021.705803

**Published:** 2021-06-18

**Authors:** Kejun Wang, Lige Zhang, Dongdong Duan, Ruimin Qiao, Xiuling Li, Xinjian Li, Xuelei Han

**Affiliations:** College of Animal Sciences and Technology, Henan Agricultural University, Zhengzhou, China

**Keywords:** pig, whole-genome resequencing, introgression, selection, genetic

## Abstract

Introgression of genetic features from European pigs into Chinese pigs was reported possibly contributing to improvements in productivity traits, such as feed conversion efficiency and body size. However, the genomic differences from European pigs and the potential role of introgression in Henan indigenous pigs remains unclear. In this study, we found significant introgression from European pigs into the genome of Chinese indigenous pigs, especially in Henan indigenous pigs. The introgression in Henan indigenous pigs, particularly in the Nanyang black pig, was mainly derived from Duroc pigs. Most importantly, we found that the *NR6A1, GPD2*, and *CSRNP3* genes were introgressed and reshaped by artificial selection, and these may have contributed to increases in pig body size and feed conversion efficiency. Our results suggest that human-mediated introgression and selection have reshaped the genome of Henan pigs and improved several of their desired traits. These findings contribute to our understanding of the history of Henan indigenous pigs and provide insights into the genetic mechanisms affecting economically important traits in pig populations.

## Introduction

Livestock animals were domesticated from wild ancestors for human use, subsequently undergoing contiguous breeding and improvement through the generation of breeds with distinct phenotypes. Crossbreeding between breeds or subspecies is often conducted during the breeding process to improve livestock's productivity or desired appearance (Bosse et al., 2014). Examples of this selective breeding includes yellow skin, penciled feathers, and spotted comb in domesticated chickens resulting from introgressions from *Gallus sonneratii, Gallus varius*, and *Gallus lafayettii* (Morejohn, 1968; Eriksson et al., 2008; Fallahshahroudi et al., 2019). Human-mediated introgression of alleles plays an important role in altering the genome of domesticated animals, including pigs (Bosse et al., 2014).

Divergence between Asian wild and European wild pigs can been traced to approximately half a million years ago, prior to independent domestication (~10,000 years ago) (Giuffra et al., 2000; Larson et al., 2005; Groenen et al., 2012). Subsequently, many domesticated pig populations were formed, each with distinct phenotypes. Since then, hybridization and introgression have reshaped the modern pig genome (Giuffra et al., 2000; Goedbloed et al., 2013). According to historical records, the Chinese pig was imported into Europe in the mid-to-late 18th century (Chen et al., 2020). This introgression was likely to have played an important role in improving the reproductive traits of the Large White (LW) pig (Chen et al., 2020). Moreover, Chinese pigs were imported into America to improve the production performance of American indigenous pigs through crossbreeding, and this accounts for about 33% of the genome on average (Fang and Andersson, 2006). Several studies based on genetic data have revealed evidence for introgression between sub-populations (Yang et al., 2009, 2014; Jiang et al., 2012; Wang et al., 2020). However, the genomic differences and functions introduced by introgression are still not well understood. Henan is a central province of China with a rich diversity of indigenous pig breeds. Three indigenous pig breeds, the Nanyang Black pig (NY), the Huainan pig (HN), and the Queshan black pig (QS), have been named based on the classification scheme from the National Commission of Animal Genetics resource book (2011). These three pig breeds exhibited strong disease-resistance, high meat yield and early maturation (Qiao et al., 2019). Low density SNP analysis revealed that there has been admixture between Henan indigenous pigs and European commercial pigs (Qiao et al., 2019). However, the genomic differences and potential role of introgression in Henan indigenous pigs is still unclear.

Here, whole genome data of Henan indigenous pigs were generated and analyzed together with data for European pigs and other Chinese indigenous pigs. Furthermore, admixture and population structures were investigated. Subsequently, introgression from European pigs into Henan indigenous pigs was determined by multiple statistical analyses, and the proportion of introgression was estimated. Most importantly, common introgressed regions and selection regions were identified, which were possibly associated with high feed conversion efficiency and larger body size.

## Results and Discussion

### Whole-Genome Resequencing of Henan Indigenous Pigs

A set of whole-genome sequencing data comprising 102 individuals was collected, from nine Chinese indigenous pig breeds (CD), four European commercial pig breeds (EUD), one Chinese wild pig population (CW), one European wild pig population (EUW), and one Java warty pig (*Sus verrucosus*, an outgroup) (Supplementary Table 1). Of these, we sequenced 30 pigs from three Henan indigenous pigs (CDh), generating whole-genome data with an average ~15x sequencing depth. Approximately 66.3 million autosomal biallelic SNPs were identified. After filtering minor allelic frequencies <0.01 and call rates >0.9, about 29.9 million autosomal biallelic SNPs were ultimately retained and used in subsequent analyses.

### Inference of Population Structure

**Figure 1:**
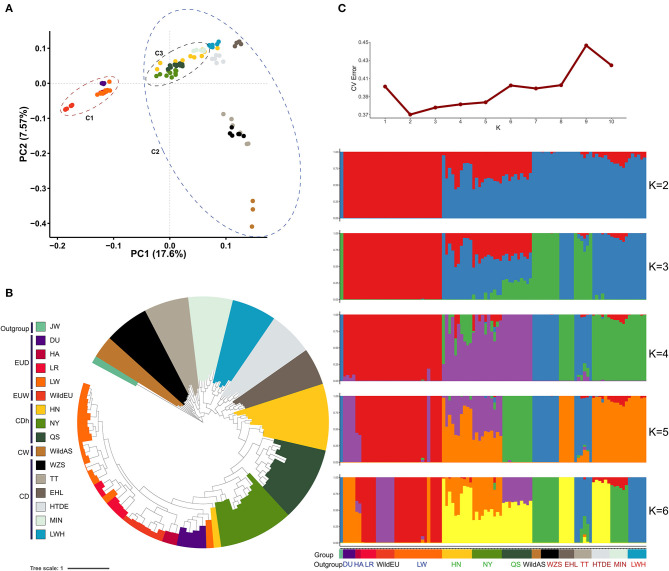
Population structure and relationships. **(A)** Principal component analysis of Chinese and European pigs. C1, European pigs; C2, Chinese pigs; C3, Henan indigenous pigs. **(B)** Phylogenetic tree of Chinese and European pigs based on the GTR model. Group colors used in **(A)** are the same as **(B)**. **(C)** Population structure of Chinese and European pigs revealed by admixture analysis. JW, Java warty pig; DU, Duroc pig; HA, Hampshire pig; LR, Landrace pig; LW, Large White pig; WildEU, European Wild pig; HN, Huainan pig; NY, Nanyang pig; QS, Queshan pig; WildAS, Chinese wild pig; WZS, Wuzhishan pig; TT, Tibetan pig; EHL, Erhualian pig; HTDE, Hetao daer pig; MIN, Min pig; and LWH, Laiwuhei pig. All pigs were also classified as Chinese indigenous pig breeds (CD), European commercial pig breeds (EUD), Chinese wild pig populations (CW), European wild pigs (EUW), or Java warty pigs (*Sus verrucosus*, the outgroup).

Divergence between Asian wild and European wild pigs was traced to approximately half a million years prior to subsequent independent domestication (~10,000 years ago) (Giuffra et al., 2000; Larson et al., 2005). Evidence from principal component analysis (PCA) revealed obvious differentiation between Chinese pigs and European pigs, representing the genetic differences between these sub-species (Figure 1A). The first principal component (PC1) captured 17.6% of the total eigenvalue, dividing individuals into two clades (European pigs and Chinese pigs). The second principal component (PC2) captured 7.57% of the total eigenvalue, dividing Chinese pigs into Southwestern indigenous pigs (the Tibetan, TT, and Wuzhishan pigs, WZS), Chinese wild pigs, and other indigenous pigs. Interestingly, we found that a cluster of three Henan indigenous pig (CDh) breeds showed a closer relatedness to European pigs (Figure 1A). When rooting using the Java warty pig (*Sus verrucosus*), a phylogenetic tree based on a GTR model demonstrated that different subgroups formed obvious clades (Figure 1B). Interestingly, we also observed that Henan indigenous pigs (CDh) clustered with European pigs. These results imply admixture between Henan indigenous pigs (CDh) and European pigs. According to the methods found in Evanno et al. 2005, the best estimate for the number of clusters (*K*-value) was equal to 2 (Figure 1C). When *K* = 2 in the admixture graph, two ancestral lineages separated Chinese pigs and European pigs from the population in which admixture occurred (Figure 1C). Obviously, CDh and two CD pigs (HTDE and MIN) showed significant admixture with a European lineage, especially in Nanyang pigs (NY). Collectively, these findings revealed the clear signature of admixture of Chinese indigenous pigs with European pigs, especially in Henan indigenous pigs (CDh). This result was also consistent with previous evidence of European pig introgression with MIN pigs (MIN), Laiwu black pigs (LWH), Hetao pigs (HTDE), and Tibetan pigs (TT) (Chen et al., 2020; Wang et al., 2020).

### Gene Flow of Duroc Pigs to Henan Indigenous Pigs

**Figure 2:**
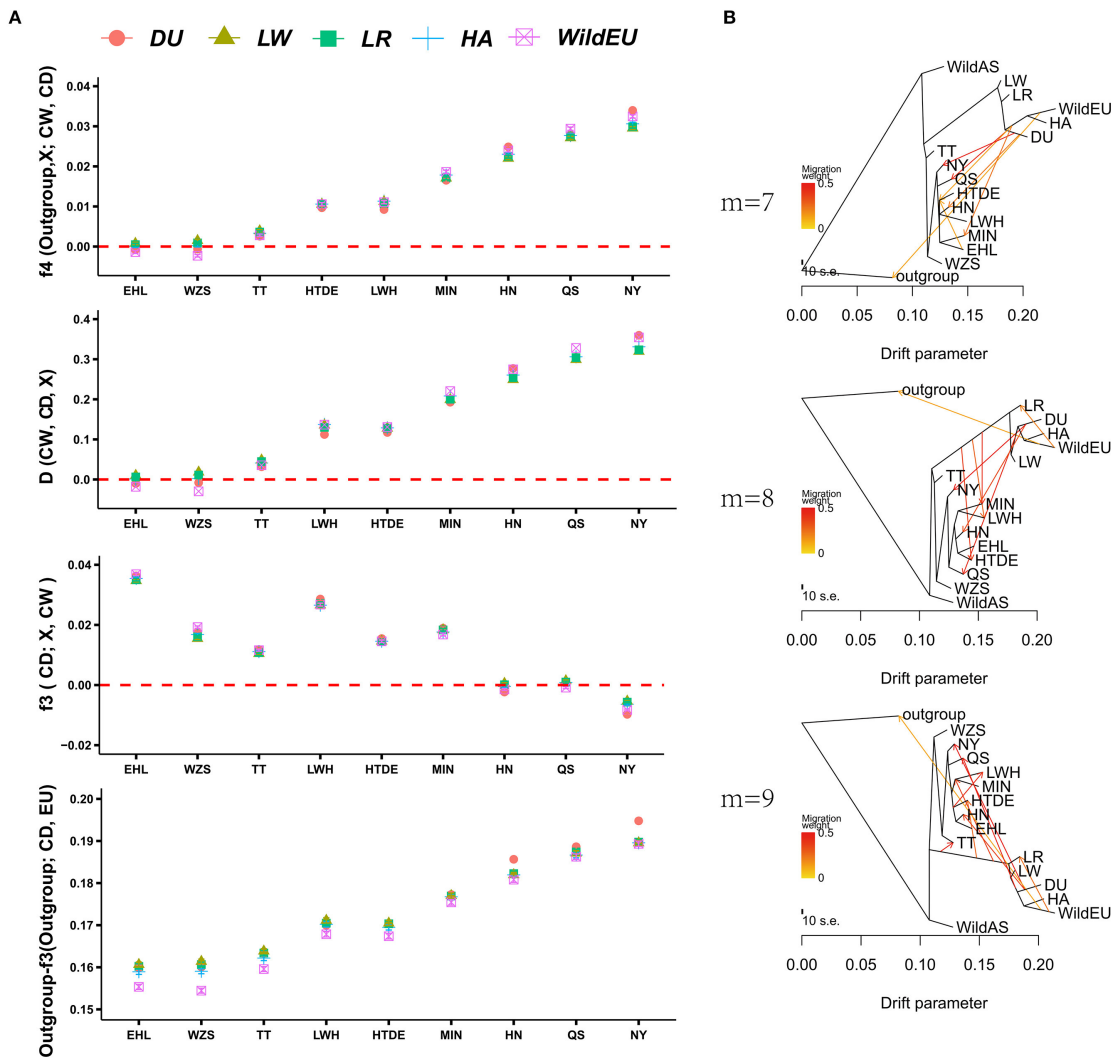
Admixture of Chinese indigenous pigs and European pigs. **(A)** Evidence from *f4* statistics, *D-statistics, f3* statistics, and *outgroup-f3* statistics showing admixture of Chinese and European pigs. **(B)** TreeMix analysis revealing gene flow from European to Chinese pigs.

To obtain direct introgression evidence, *f4* statistics and *D-statistics* were calculated for each combination of European and Chinese indigenous pigs. As expected, we found evident gene flow of European pigs in seven Chinese indigenous pig breeds according to *f4* statistics, with the exceptions of WZS and EHL pigs (Figure 2A, Supplementary Figure 1). The order of introgression intensity from high to low was NY > QS > HN > MIN > HTDE > LWH > TT. Evidence from *D-statistics* showed the same pattern as *f4* statistics did. We also calculated *f3* statistics (CD; European pig, CW) to validate the admixture of Chinese indigenous pigs from European pigs and Chinese wild pigs. We only observed significant admixture for NY pigs, whereas HN and QS pigs were close to the threshold value (Figure 2, Supplementary Figure 1). This implied that *f3* statistics (CD, CW, European pigs) were insensitive to introgression at low proportions. To identify which breed of European pig was responsible for the introgression detected, the outgroup*-f3* statistics were explored. The Duroc (DU) pig was more genetically similar to the NY, QS, and HN pigs than to European pigs, while LW pigs were closer to other Chinese indigenous pigs. This result might be explained by historical evidence indicating that Chinese pigs were introduced into European breeds to improve the fertility and immunity of LW pigs (Chen et al., 2020). Alternatively, introgression of LW into some Chinese indigenous pigs could not be ruled out.

To obtain more detailed introgression evidence, TreeMix analysis was conducted. The TreeMix analysis presented the same results as above, i.e., that admixture and division between European and Chinese pigs had occurred (Figure 2B). Increasing the number of migration events marginally enhanced the fitness of the model testing. When *m* = 7, the model explained 0.9936 of the variance for the autosomal SNP data, while when *m* = 8 or 9, the model explained 0.9957 or 0.9963 of the variance, respectively. Evidence from TreeMix also indicated significant gene flow from DU into CDh (HN, QS, and NY), consistent with the results of our outgroup*-f3* analysis. We also observed weak introgression from DU to MIN, HTDE, TT, and LWH, which was consistent with the previous introgression results based on SNP chip data (Wang et al., 2020).

### Identification of Introgression and Selective Regions in Henan Indigenous Pigs

**Figure 3:**
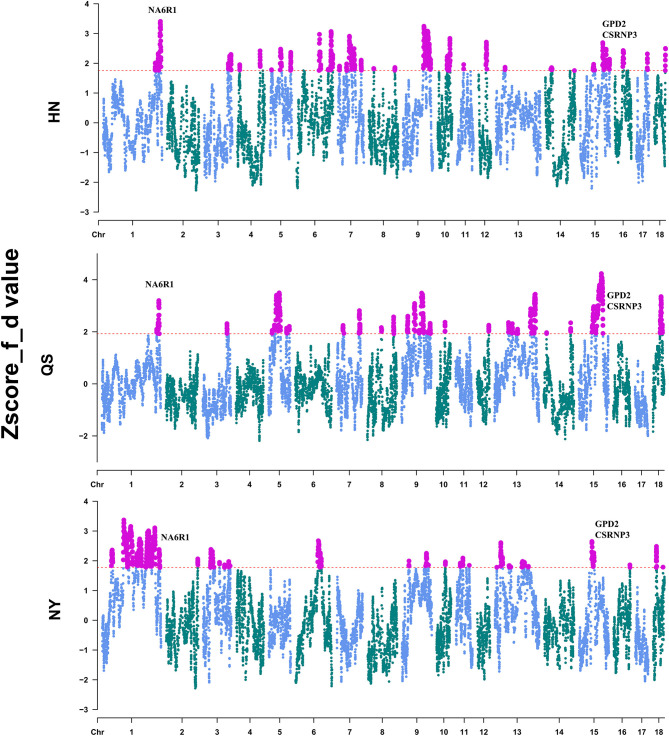
Identification of the introgression region of Duroc pigs to Henan indigenous pigs. The red dashed line shows the 5% cutoff used to define the introgression region.

To locate the detailed genomic regions for the gene flow from DU to CDh, we calculated the f-statistic (*f_d*) value for each combination of DU and CDh (HN, QS, and NY) by the sliding-window method. After Z-transformation, the top 5% of the windows were considered to be outlier introgression regions (Figure 3, Supplementary Table 2). Totals of 506, 487, and 522 regions passed this threshold (Supplementary Table 2). Combining the overlapping regions, these identified introgression regions accounted for 12.53% of NY, 10.61% of HN, and 9.43% of the QS genome. The overlapping regions of CDh were located on three chromosomes, comprised of chr1:262,974,058-267,766,091, chr9:123,030,904-123,611,236, and chr15:61,503,802-74,093,823. Genes located in these overlapped regions were retrieved from the Ensemble database and are shown in Supplementary Table 3. Of these, the *NR6A1, CSRNP3*, and *GPD2* genes were considered to be putative important introgression genes (Figure 3).

To assess the importance of domestication and post-domestication selection on Henan indigenous pigs in the face of gene flow, we conducted a whole-genome scan for selective signatures via computing the XP-CLR of each CDh relative to Chinese wild boars (Supplementary Figure 2). A total of 342 selective regions were commonly identified in these three CDh (Supplementary Table 4). Genes located in the selective regions are shown in Supplementary Table 5. Two of three introgression regions were identified as being under selection and were divided into seven small genome regions: (chr1: 264,350,001–264,500,000, chr1: 265,300,001–265,600,000, chr1: 266,150,001–266,250,000, chr15: 63,100,001–63,250,000, chr15: 63,700,001–63,800,000, chr15: 64,600,001–64,700,000, and chr15: 72,100,001–72,200,000). A total of 10 coding genes and 2 non-coding genes were included, in these regions, consisting of *NR6A1, NR5A1, MAPKAP1, OLFML2A, DENND1A, CSRNP3, GPD2, ENSSSCG00000042870, ENSSSCG00000047320, ENSSSCG00000043788, ssc-miR-181a-2*, and *ssc-miR-181b-2*.

### Introgression and Selection Reshaped the *NR6A1* Locus of Henan Indigenous Pigs

**Figure 4:**
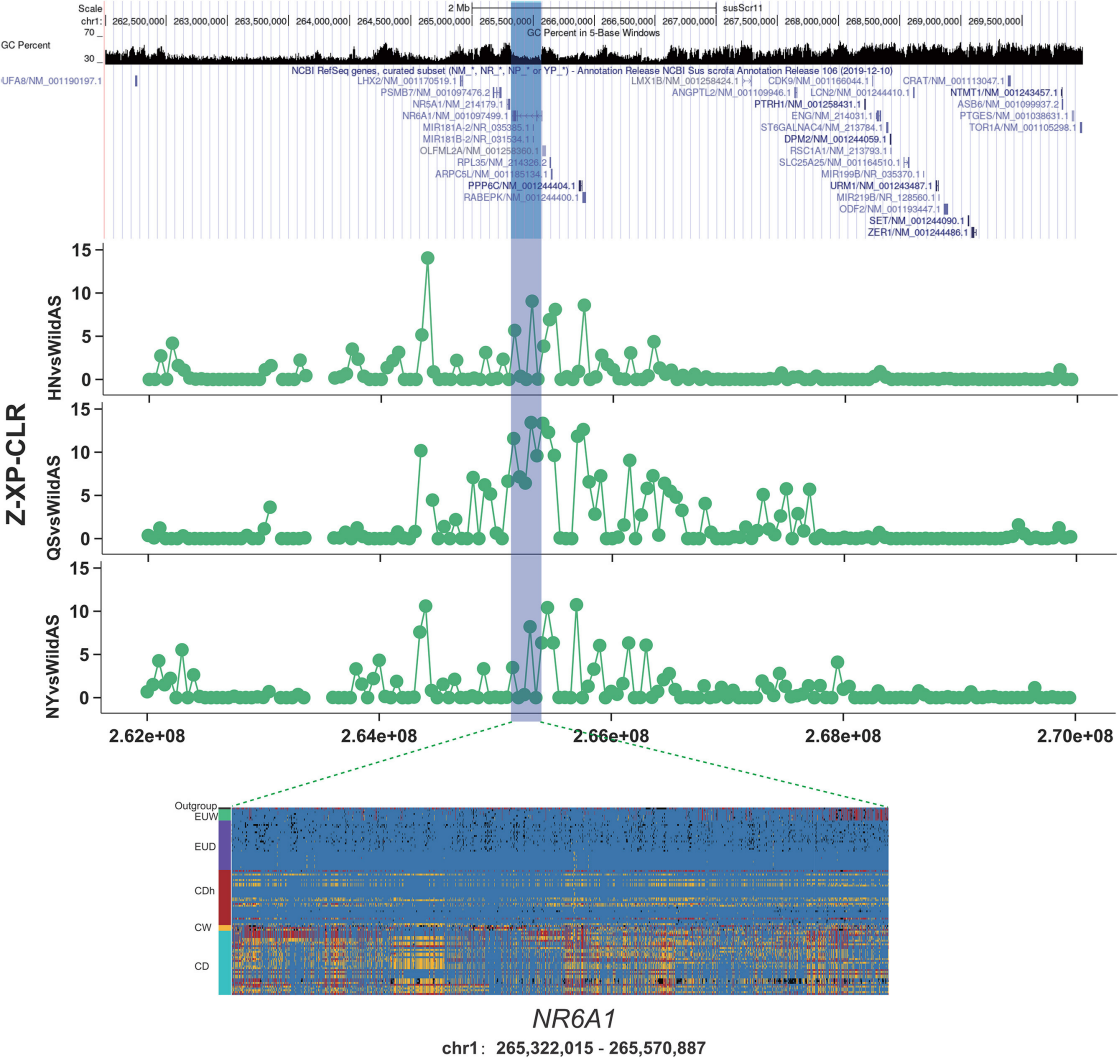
Introgression at the *NR6A1* locus. XP-CLR showed a significant selection signature located in the *NR6A1* gene in Henan indigenous pigs compared to Chinese wild pigs. Results from genotyping a diverse panel of European and Chinese pigs for SNPs in the putative selective sweeps containing *NR6A1*.

The *NR6A1* gene, nuclear receptor subfamily six group A member 1, encodes an orphan nuclear receptor. It has been widely reported to have been under selection during domestication and was shown to be associated with the number of vertebrae (Ribani et al., 2019; Zhang et al., 2019, 2020). A variant allele of p.Pro192Leu of *NR6A1* was correlated with an increase in the number of pig thoracic and dorsal vertebrae, from an average of 19 to 21–23 (Mikawa et al., 2007). An increased number of vertebrae plays a positive role in increasing body length and overall meat production (Burgos et al., 2015). Variation in the *NR6A1* gene is under selection and nearly fixed in European commercial pigs relative to European wild boars (Rubin et al., 2012). This implies the positive effect of the *NR6A1* gene was associated with this human desired production trait. In this study, we found that a region encompassing the *NR6A1* gene was significantly introgressed in Henan indigenous pigs from Duroc pigs based on *f_d* values (Figure 3). Moreover, we observed a remarkably high XP-CLR value in CDh pigs relative to CD pigs (Figure 4). Results from genotyping of European pigs and Chinese pigs for SNPs in the *NR6A1* region also showed that CDh pigs showed significant differences from Chinese pigs and greater similarity to EUD pigs. This region was reported to be fixed in European commercial pigs relative to European wild pigs and Chinese pigs represented by Meishan pigs (Rubin et al., 2012). Additionally, the *NR6A1* locus in European pigs was also reported to have introgressed into Chinese Licha pigs and has been associated with increasing vertebral number (Yang et al., 2009). Therefore, it can be inferred that this region was first introgressed into Henan indigenous pigs from European commercial pigs and was then subjected to conscious or unconscious artificial selection, since the *NR6A1* gene functions in increasing body length and meat production that are human-desired traits.

### Introgression and Selection Reshaped the *GPD2* and *CSRNP3* Loci of Henan Indigenous Pigs

**Figure 5:**
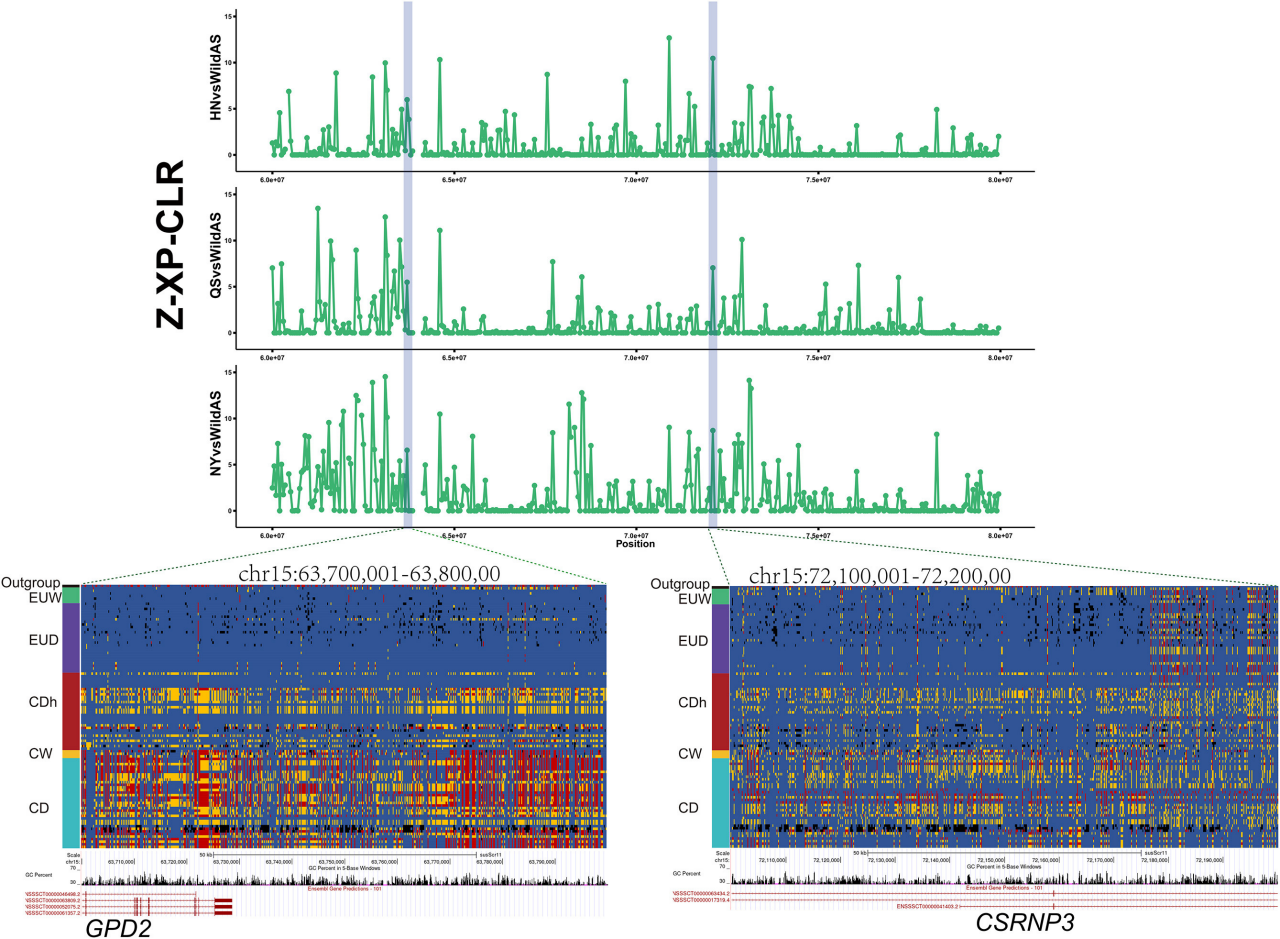
Introgression at the *GPD2* and *CSRNP3* loci. XP-CLR showed significant selection signatures located in the *GPD2* and *CSRNP3* genes in Henan indigenous pigs compared to Chinese wild pigs. Results from genotyping a diverse panel of European and Chinese pigs for SNPs in the putative selective sweeps containing *GPD2* or *CSRNP3*.

European commercial pigs demonstrated significantly better production performance than Chinese indigenous pigs in traits such as higher feed conversion efficiency and larger body size. In this study, we found that the loci *GPD2* and *CSRNP3* were introgressed from Duroc pigs into three Henan indigenous pig breeds (Figure 3). Moreover, these regions presented significant selection signals in Henan indigenous pig breeds relative to Chinese wild pigs (Figure 5). *GPD2*, encoding glycerol-3-phosphate dehydrogenase, catalyzes the unidirectional conversion of glycerol-3-phosphate to dihydroxyacetone phosphate and eventually to glycolysis (Gerbitz et al., 1996). The *GPD2* gene was shown to be significantly associated with residual feed intake traits, and this locus was significantly down-regulated in individuals with lower residual feed intake relative to those with higher residual feed intake (Lkhagvadorj et al., 2010). *CSRNP3* encodes a transcription factor, cysteine and serine rich nuclear protein 3, and the expression level of *CSRNP3* was associated with pig residual feed intake and feed conversion ratio traits (Vincent et al., 2015; Messad et al., 2019). In cattle, SNPs located in *CSRNP3* were significantly associated with chest width, hip width, or rump width (Doyle et al., 2020). We also compared these genotypes between European and Chinese pigs, and these showed obvious similarity to European commercial pigs (EUD) compared to Chinese wild pigs and other Chinese indigenous pigs (Figure 5). As is well-known, European commercial pigs have a higher feed conversion efficiency and a larger body size than Chinese native pigs. Functions of the *CSRNP3* and *GPD2* genes in feed conversion efficiency and body size may have driven the alleles from European commercial pigs to near fixation after introgression into Henan indigenous pigs under human subjective selection.

## Conclusions

Collectively, we found significant introgression from European pigs into Chinese indigenous pigs, especially in Henan indigenous pigs. The introgression derived from Duroc pigs was captured for Henan indigenous pigs, especially in Nanyang black pigs. Most importantly, we found that the *NR6A1, GPD2*, and *CSRNP3* genes were introgressed and selected factors that may contribute to pig body size and feed conversion ratio traits. This suggests that human-mediated introgression and selection could have reshaped the pig genome and improved several desired traits. These findings further deepen our understanding of the history of Henan indigenous pigs and provide insights into the genetic mechanisms affecting economically important pig traits.

## Materials and Methods

### Ethics Statement

Ear tissues of three indigenous pigs from Henan province were collected in strict accordance with protocols approved by the Institutional Animal Care and Use Committee (IACUC) at Henan Agricultural University.

### Sample Collection

Genome resequencing data for 102 pigs was collected in this study, of which 30 individuals were derived from three Henan indigenous breeds and 10 Yorkshire pigs were generated in our previous study (Li et al., 2020). We downloaded 68 accessions chosen from recent studies from the National Center for Biotechnology Information (NCBI) Sequence Read Archive database, which included from 13 breeds (Supplementary Table 1). Multiple statistics were used to improve the accuracy of the results in this study, but we could not rule out sampling error caused by differences in sample size.

### Whole-Genome Resequencing and SNP Calling

The procedures for DNA extraction and library construction were described in our previous studies (Li et al., 2020; Shang et al., 2020). Paired end reads were generated using the BGISEQ-500 platform (BGI Genomics Co., Ltd.). Raw reads were filtered using the Trimmomatic software (seed mismatches: palindrome: simple clip −2:30:10) (Bolger et al., 2014). Filtered reads were aligned to a reference genome (Sscrofa v11.1) using the Burrows–Wheeler alignment (BWA) tool with default parameters (Li and Durbin, 2009). SAM files were merged and sorted using SAMtools (Li et al., 2009). Subsequently, duplicate reads were marked using the MarkDuplicates function of Picard Tools (http://broadinstitute.github.io/picard/). SNP calling for each individual was implemented using the HaplotypeCaller program of the GATK4 software and further joint calling using the GenomicsDBImport and GenotypeGVCFs programs of GATK4 (v4.1.2.0) (McKenna et al., 2010). Finally, we filtered our SNP panel using the Variant Filtration program of GATK4 with the parameters “*QD*<*2.0 || FS* > *200.0 || SOR* > *10.0 || MQRankSum* < –*12.5 || ReadPosRankSum* < –*8.0*.”

### Population Structure Analysis

Biallelic SNPs located on autosomes were identified and retained using the VCFtools software (0.1.16) (http://vcftools.github.io/) (Danecek et al., 2011). Subsequently, variants with a call rate >90% and MAF >0.01 were identified and retained (http://www.cog-genomics.org/plink2/) using PLINK (v1.9) (Chang et al., 2015). Principal Component Analysis (PCA) was also performed using PLINK. Scatter distributions for each individual were plotted using the first principal component. Population assignment analysis was conducted using the Admixture software (Kalyaanamoorthy et al., 2017), and the best number of clusters (K) was determined by the method from Evanno et al. (2005). A phylogenetic tree was constructed using the IQ-TREE software (v 1.6.12) with 1,000 bootstrap replicates based on the GTR model (Kalyaanamoorthy et al., 2017) and visualized using the iTOL online web server with a root at the outgroup (Letunic and Bork, 2019). The Java warty pig (*Sus verrucosus*) was defined as the outgroup for these analyses.

### Whole-Genome Analysis of Genomic Introgression

To investigate the admixture and splitting of domesticated pigs, the separation and admixture were inferred using the TreeMix software (v. 1.13) (Pickrell and Pritchard, 2012). The *threepop* and *fourpop* programs of TreeMix were used to identify the introgression from European pigs to Chinese pigs. The *f4* (outgroup, EUD; CW, and CD) statistics were calculated to measure the shared drift between European and CD (Chinese domestic pigs) based on allelic frequency (Patterson et al., 2012). For the *f4* statistics, positive values indicated gene flow between European pigs and CD pigs under the hypothesis that there was no admixture of the outgroup into any group. The Java warty pig (*Sus verrucosus*) was defined as the outgroup in this study. The *outgroup-f3* (outgroup; CW, EUD) statistics measured the branch lengths from the outgroup to the common ancestor of Chinese domestic pigs and European pigs, and higher values indicated greater genetic similarity. Another *f3* statistic, *f3* (CD; CW, ED), was computed to identify the admixture of CD with CW and EUD pigs. The pattern's *D-statistic* was also computed using the Dsuite software (Patterson et al., 2012). The *f_d* statistics for each window were also computed to investigate the introgression region with sliding windows containing 10,000 SNP and 1,000 SNP steps. A Z-transformation of *f_d* statistics was treated using the “scale” function in R, of which the top 5% were defined as significant introgression regions. Genes located within the introgression regions were retrieved using the Biomart program of the ENSEMBLE web server.

### Signatures of Selection

Selection signatures across the whole genome were scanned in a comparison between Henan indigenous pigs and Chinese wild pigs. The XP-CLR (cross-population composite likelihood ratio) was calculated using a 100-kb sliding window with a step size of 50 kb (https://github.com/hardingnj/xpclr). Usually, a genetic distance of one cM is empirically equal to one Mb of physical distance. The regions with top 5% normalized xp-clr values were classified as significantly selected between each comparison. Genes located within the selective regions were retrieved using the Biomart program of the ENSEMBLE web server.

## Data Availability Statement

The datasets presented in this study can be found in online repositories. The names of the repository/repositories and accession number(s) can be found at: https://bigd.big.ac.cn, CRA004121.

## Author Contributions

KW, XinL, and XH participated in the design of the study and obtained project funding. KW, XinL, XH, LZ, DD, RQ, and XiuL collected the samples. KW carried out the analysis, interpretation of data, and initiated and drafted and revised the manuscript. XinL and XH revised the manuscript. All of the authors have read and approved the final manuscript.

## Conflict of Interest

The authors declare that the research was conducted in the absence of any commercial or financial relationships that could be construed as a potential conflict of interest.

## Supplementary Material

Supplementary Figure 1 Z-transformation of f4 (outgroup, X; CW, CD) and f3 (outgroup; CW, CD). X indicates one of the European pig breeds.

Supplementary Figure 2 Z-XP-CLR values calculated for each combination between Henan indigenous pigs (CDh) and Chinese wild pigs (CW). The red dashed lines represent the 5% cutoff values used to define selective signatures.

Supplementary Table 1 Sample information used in this study.

Supplementary Table 2 Introgression genomic regions in Henan indigenous pigs from Duroc pigs.

Supplementary Table 3 Genes located in the common regions of introgression from Duroc to Henan indigenous pigs.

Supplementary Table 4 Common significantly selective regions of Henan indigenous pigs.

Supplementary Table 5 Genes located in the common selective regions of Henan indigenous pigs.
