# Supplementary figures and images for: Genomic Analysis Reveals Human-Mediated Introgression From European Commercial Pigs to Henan Indigenous Pigs
Source: Front Genet. 2021 Jun 18;12:705803. doi: 10.3389/fgene.2021.705803 (PMC8249855; doi:10.3389/fgene.2021.705803)

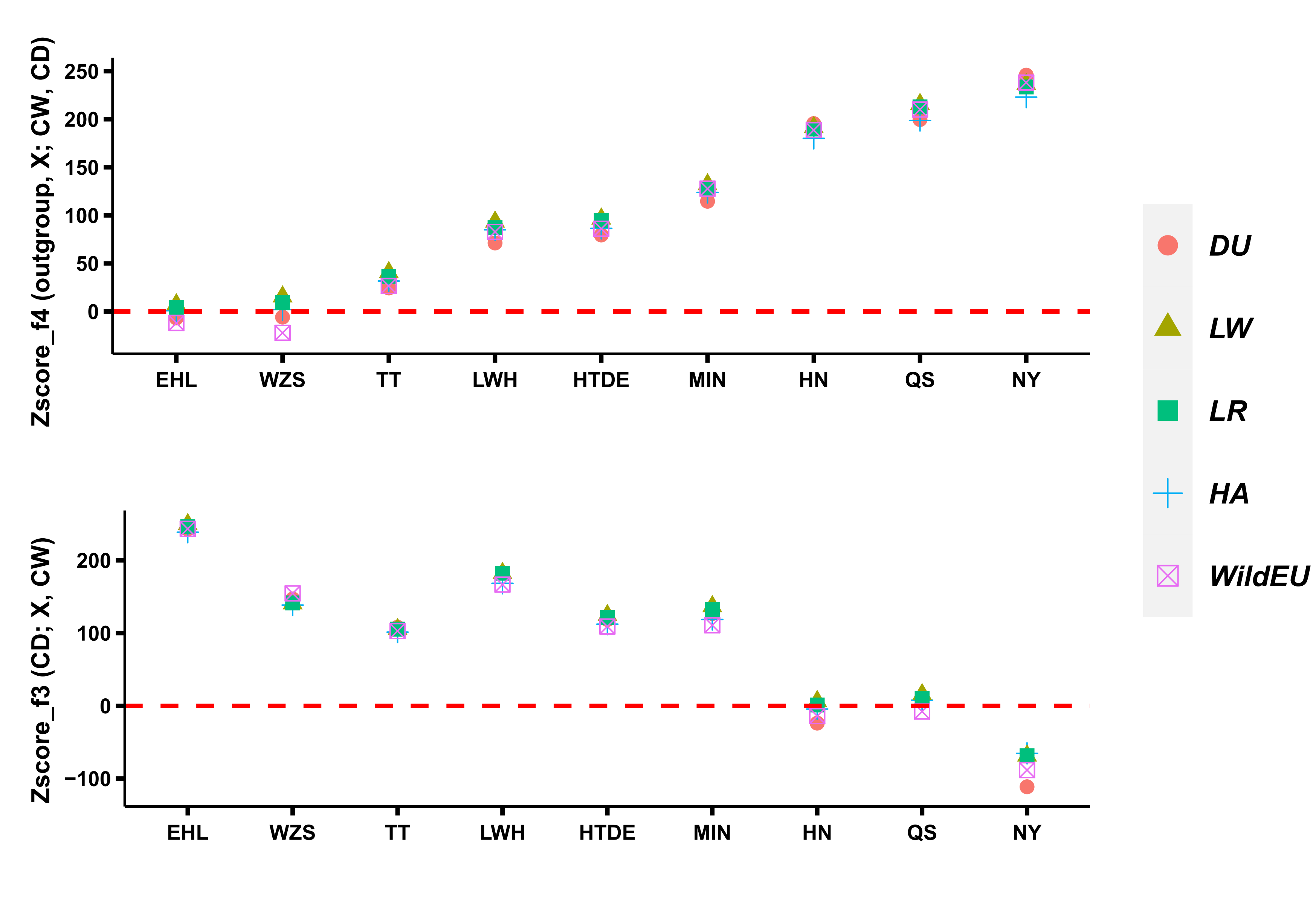

Supplement: Supplementary Figure 1 — Z-transformation of f4 (outgroup, X; CW, CD) and f3 (outgroup; CW, CD). X indicates one of the European pig breeds. [file Image_1.TIF]

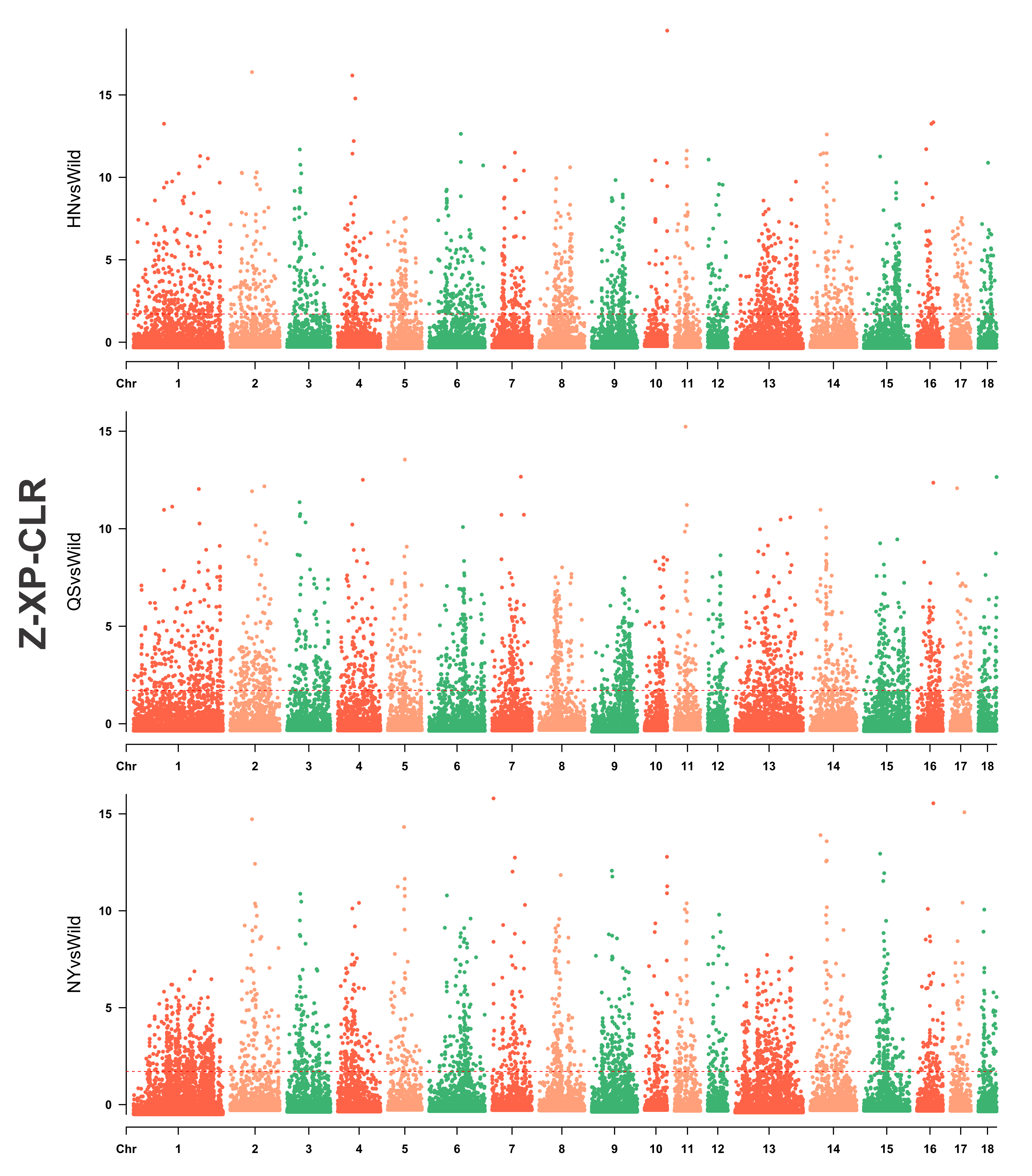

Supplement: Supplementary Figure 2 — Z-XP-CLR values calculated for each combination between Henan indigenous pigs (CDh) and Chinese wild pigs (CW). The red dashed lines represent the 5% cutoff values used to define selective signatures. [file Image_2.TIF]
